# Reduced expression of central innate defense molecules in pancreatic biopsies from subjects with Type  1 diabetes

**DOI:** 10.1007/s00592-024-02286-1

**Published:** 2024-05-08

**Authors:** Angie Tegehall, Sofie Ingvast, Lars Krogvold, Knut Dahl-Jørgensen, Olle Korsgren

**Affiliations:** 1 Rudbeck Laboratory, Department of Immunology, Genetics and Pathology, Uppsala University, 751 85 Uppsala, Sweden; 2 Division of Paediatric and Adolescent Medicine, Oslo University Hospital, Oslo, Norway; 3 Institute of Clinical Medicine, Faculty of Medicine, University of Oslo, Oslo, Norway

**Keywords:** Biopsy, Defensins, Etiopathology, Innate immunity, Pancreas, Type 1 diabetes

## Abstract

**Aims/Hypothesis:**

Defensins play a crucial role in the innate immune system's first defense against microbial threats. However, little is known about the defensin system in the pancreas, especially in relation to Type 1 diabetes. We explore the expression of defensins in different disease stages of Type 1 diabetes and correlated obtained findings to the degree of inflammation, providing new insights into the disease and the innate immune system.

**Material and methods:**

Pancreases from non-diabetic human organ donors of different age groups and donors with Type 1 diabetes with different disease duration were examined. Sections from head, body and tail of the pancreas were stained for eight different defensins and for immune cells; CD3+, CD45+, CD68+ and NES+ (granulocytes).

**Results:**

In non-diabetic adult controls the level of expression for defensins Beta-1,Alpha-1, Cathelicidin and REG3A correlated with the level of inflammation. In contrast, individuals with Type  1 diabetes exhibit a reduction or absence of several central defensins regardless of the level of inflammation in their pancreas. The expression of Cathelicidin is present in neutrophils and macrophages but not in T-cells in subjects with Type 1 diabetes.

**Conclusions:**

Obtained findings suggest a pancreatic dysfunction in the innate immune system and the bridging to the adaptive system in Type 1 diabetes. Further studies on the role of the local innate immune system in Type 1 diabetes is needed.

## Introduction

Defensins are small molecules (2–5 kDa) that play an important role in the innate and adaptive defence system. They belong to a large family of antimicrobial peptides of ancient conservation. Humans express two subfamilies, Alpha- and Beta-defensins which differ in the length of cysteine peptide segments and pairing of the cysteines by disulphide bonds [1–3]. The Alpha family is most often regulated on the secretory level by release from granules in neutrophils or Paneth cells upon a trigger in the environment, while the Beta family is regulated transcriptionally [1, 4, 5]. Epithelial cells in various tissues consistently express certain Beta-defensins, such as hbD-1 [5, 6]. Inflammation or exposure to bacterial toxins in the environment can lead to modulation and increase of Beta-defensins [6, 7].

Defensin families differ in structure and regulation, they also differ in their antimicrobial action and modulation of the immune system. Beta-defensins have antimicrobial, anti-viral and chemotactic properties [8] and are considered to have a role in the adaptive immune response. The antimicrobial effect has predominantly been shown against gram negative bacteria, such as *Escherichia coli* [9]. Alpha-defensins exhibit potent antimicrobial activity against both gram-negative and gram-positive bacteria by membrane disruption. Additionally, they have been shown to inhibit the adhesion of enveloped viruses to host cells and prevent uncoating of capsid viruses [5]. Thus far, there has been no description of a link to the adaptive system for the Alpha-defensin family.

Defensins can also drive an inflammatory process into a chronic pathological state involving other inflammatory mediators and the adaptive immune system [10]. Psoriasis and Crohn's disease are two clinically significant examples where altered defensin expression plays an important role [11].

Although defensins have been extensively researched in the skin, gut, and oral cavity, their function in the pancreas remains largely unknown [12, 13]. Several studies describe ongoing inflammation of the pancreas in subjects with recent onset Type 1 diabetes [14–17]. There is also an association between bacterial infections during infancy and a high risk of developing islet-autoantibodies and Type  1 diabetes [18]. Translocation of bacteria and viruses from the duodenum to the pancreas could trigger the activation of innate inflammatory immune response [17, 19]. The inflammatory response driven by infectious agents has been suggested as triggers for Type 1  diabetes [20, 21]. The aim of this study was to characterize the expression of different defensins in non-diabetic organ donors of different ages as well as in donors with Type 1 diabetes with different disease duration to examine a tentative role in Type 1 diabetes.

## Materials and methods

### Ethics

The research work conducted using human tissue followed guidelines outlined in the Declaration of Helsinki. Pancreatic tissue was procured from organ donors, and consent to use it for research purposes was obtained from the next of kin either verbally by the attending physician or from an online database. Procedures were fully documented according to Swedish law and regional standards. The present study utilized pancreatic samples from patients who were part of the DiViD study, approved by The Norwegian Governments Regional Ethics Committee. Prior to participation, patients were provided comprehensive oral and written information from the diabetologist and surgeon separately.

### Human pancreatic samples

Biopsies from 41 human pancreases were included in the study, divided into five different groups. One donor died at onset, four living patients (DiViD-study) [22], 13 organ donors with longstanding Type 1 diabetes, 19 non-diabetic organ donors and four organ donors under five years of age (Table 1). The adult non-diabetic individuals were matched to the subjects with Type 1 diabetes for age, sex and BMI. Three parts of the pancreas, head, body and tail, was chosen from each donor where this was possible.

**Table 1:** Detailed list of antibodies used

Antibody	Clone	Dilution	Control tissue
Anti α-1 (Abcam)	–	1:200	Human spleen
Anti β-1 Abcam)	M11-14b-D10	1:100	–
Anti β-2 (Abcam)	–	1:500	Human tonsil
Anti β-3 (Abcam)	–	1:200	–
Anti-Cathelicidin (Abcam)	–	1:1000	Human spleen
Anti-GP2 (Abcam)	GP2/1712	1:200	Human pancreas
Anti-Neutrophil-4 (Abcam)	–	1:50	Human spleen
Anti-REG3A (Abcam)	–	1:100	Human duodenum
Anti-CD45 (Agilent)	2B11 + PD7/26	1:75	Human spleen
Anti-CD68 (Agilent)	KP1	1:50	Human spleen
Anti-CD3 (Abcam)	265-3kl	1:50	Human spleen
Anti-NES (Invitrogen)	F7.2.3B	1:25	Human spleen

The organs were procured within the Nordic Network for Clinical Islet Transplantation.

### Immunohistochemistry

Formalin-fixed and paraffin-embedded tissue were cut into 6 µm sections, consecutive sections were processed and labeled using a standard immunoperoxidase technique. All antigens were unmasked by heat-induced epitope retrieval using pH 6.0 or pH 9.0 according to recommendations by the manufacturer. Primary antibodies specific for CD45, insulin, synapthofysin and eight different defensin molecules were used (Table 2). Bound antibodies were visualized using Dako EnVision or EnVision DuoFLEX Doublestain system (Agilent, California, USA) and diaminobenzidine-based substrate (Agilent, California, USA). Sections were counterstained with hematoxylin, dehydrated, mounted and analyzed by light microscope (Leica, Germany). Positive controls were running in parallel with each defensin and isotype mAbs were used as negative controls. For double staining, Cathelicidin and immune cells antibodies toward CD45, CD68, NES and CD3 were used and visualized by immunofluorescence. Confocal microscope Zeiss LSM700 (Zeiss,Germany**)** and software Zen black 3.0 SR (Zeiss, Germany) was used to analyze the slides.

**Table 2:** Donor characterization

Variable	Non-diabetic donors	Donor who died at onset of Type-1 diabetes	Donors with recent onset Type-1 diabetes	Donors with longstanding Type-1 diabetes
Number of subjects	23	1	4	13
Duration of Type-1 diabetes (weeks)	N/A	0	3–9	> 200
Age	34.5 ± 21.9	29	28.5 ± 4.8	44.8 ± 20.4
Female	10	–	2	7
Male	13	1	2	7
Body mass index (kg/m^2^)	24.3 ± 5.22	24.2	22.8 ± 2.2	25.7 ± 5.12
Hba1c	37.5 ± 3.5	95	72.75 ± 4.32	73 ± 25

*N/A* not applicable

### Analysis and statistical analysis

All slides were analyzed by two independent investigators blinded with regard to donor type. The analysis was performed as a semi-quantitative method using a standard four scale IHC score system (0,1,2,3). The IHC score was set by combining three parameters. (1) staining intensity, (2) proportion of stained pancreatic area, and (3) staining pattern. The staining was evaluated with regard to cytoplasmic and nucleic expression of the various defensins in exocrine and endocrine pancreas, as well as in blood vessels, connective tissue, ducts and adipose tissue of the pancreas. Immune cells were analyzed by comparing overlay of staining between Cathelicidin and immune cell staining.

### Statistical analysis

A mean IHC score for each donor was calculated from the examined sections.

The mean IHC score for each subject was used in the box-plot figures and statistical analysis. Statistical significance between groups were calculated by performing a Kruskal–Wallis analysis followed by Dunns multiple comparisons. The significance level was < 0.05 by using Graphpad prism 9 software.

## Results

### Beta-defensins

**Fig. 1:**
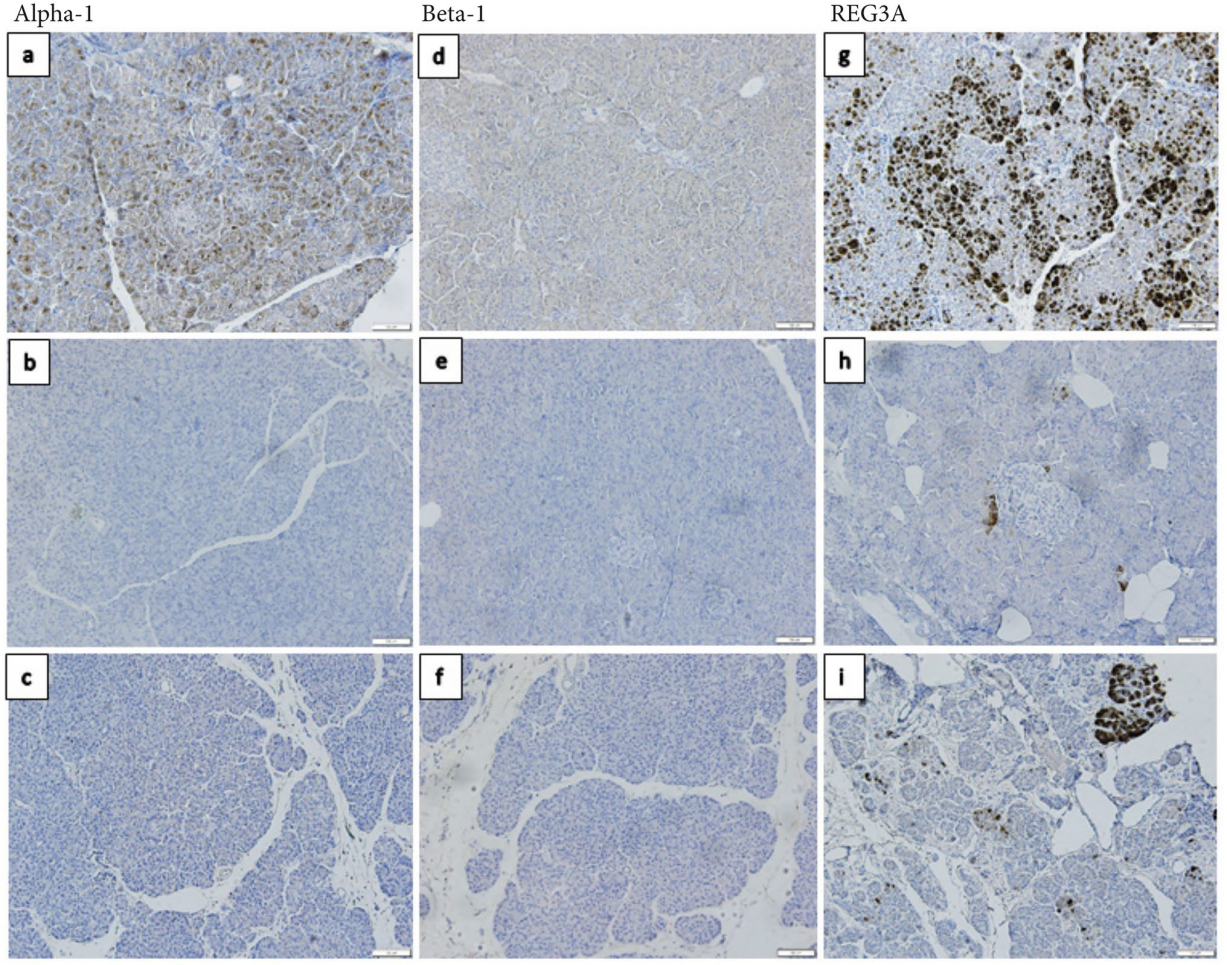
**a** Representative staining of Alpha-1 in an adult without Type 1 diabetes. A patchy cytoplasmatic staining pattern of varying intensity can be seen in the exocrine pancreas. The endocrine tissue shows low-grade positive cytoplasmatic staining. **b** Expression in a DiViD patient for Alpha-1 show negative expression in both exocrine and endocrine tissue. **c** Staining of a donor with longstanding Type 1 diabetes of defensin Alpha-1 show some areas of a low-grade cytoplasmatic staining in the exocrine pancreas and no staining in endocrine tissue. **d** Staining of Beta-1 in an adult donor without diabetes show a homogenous cytoplasmatic staining of both exocrine and endocrine tissue. **e** In a subject with recent onset Type 1 diabetes and **f** in a subject with longstanding Type 1 diabetes no expression of Beta-1 could be found in exocrine and endocrine tissue. **g** Staining of REG3A in an adult donor without diabetes show a high intensity patchy staining in the cytoplasmic compartment of the exocrine pancreas. Endocrine tissue was mostly negative. **h** Staining for defensin REG3A in DiViD patients show a low-grade patchy staining in the cytoplasmic compartment of the exocrine tissue with smaller clusters of cells with more intense staining. The endocrine tissue was negative. **i** Staining for REG3A in a donor with longstanding Type 1 diabetes show a similar staining pattern as in subjects with recent onset Type 1 diabetes but with larger areas of exocrine cells showing an intense cellular staining. Endocrine tissue was mostly negative. Original magnification × 20

**Fig. 2:**
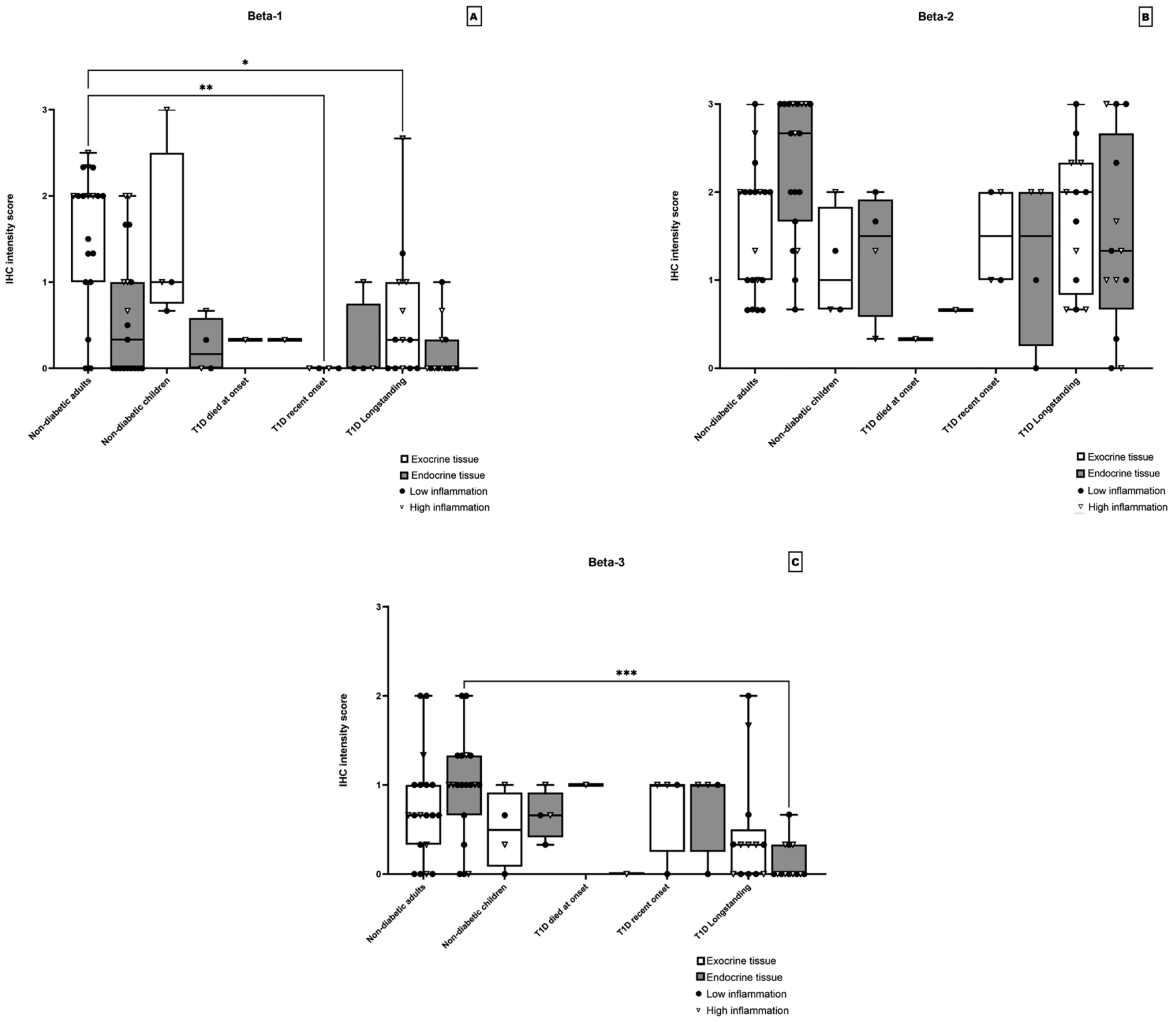
Each data point represents the mean value of the IHC score of the three pancreatic regions in one donor in endocrine and exocrine tissue, respectively. The donors were divided into different groups depending on disease status, non-diabetic adults (*n* = 19), non-diabetic children (*n* = 4), Type 1 diabetes donor that died at onset (*n* = 1), Type 1 diabetes recent onset (DiViD patients) (*n* = 4) and Type 1 diabetes longstanding donors (*n* = 13). The IHC scores for defensin Beta-1 are shown in **a**, Beta-2 in **b** and Beta-3 in **c**. Statistically significant differences are shown in each figure using Kruskal–Wallis test and Dunns test for multiple comparisons p values were non-significant > 0.05 for all groups

The three Beta-defensins had their own individual staining pattern. Beta-1 was observed as a cytoplasmic staining pattern in the exocrine compartment with varying level of expression in adult non-diabetic subjects (Fig. 1d). The most common IHC score was 0 for endocrine tissue and IHC score 2 for exocrine tissue. The same expression pattern was observed in the child group. In groups with Type 1 diabetes subjects most biopsies were negative in both endocrine and exocrine tissue (Fig. 1e, f), except for a few donors with a low-grade positive staining in the cytoplasm of the exocrine tissue (mean IHC score = 0.44). Beta-1 expression in exocrine tissue in subjects with Type 1 diabetes was significantly reduced when compared with non-diabetic subjects (*p*-value ≤ 0.05, Fig. 2a).

The staining pattern of Beta-2 displayed both a cytoplasmatic and nuclear staining with some variation in both the exocrine (mean ICH score = 1.64) and endocrine (mean ICH score = 1.83) tissue. No statistically significant difference (*p* =  > 0.05) was observed (Fig. 2b) between subjects with Type 1 diabetes and non-diabetic subjects.

Beta-3 showed a cytoplasmic expression pattern in the exocrine pancreas with little variation between groups. The endocrine tissue had an expression pattern of a low-grade cytoplasmic positive staining in biopsies from the adult non-diabetic control group, the child group and the Type 1 diabetes recent onset patients (mean IHC score=0.90). In the Type 1 diabetes longstanding donors and the donor that died at onset almost all biopsies were negative (IHC score = 0) except for a few donors with a low-grade positivity in just one part of the pancreas. In subjects with longstanding Type 1 diabetes a significant reduction in Beta-3 expression was found when compared with non-diabetic controls (*p* ≤ 0.05, Fig. 2c).

Blood vessels, connective tissue, ducts and adipose tissue were negative for the staining in all donors for all three Beta-defensins.

### Alpha-defensins

**Fig. 3:**
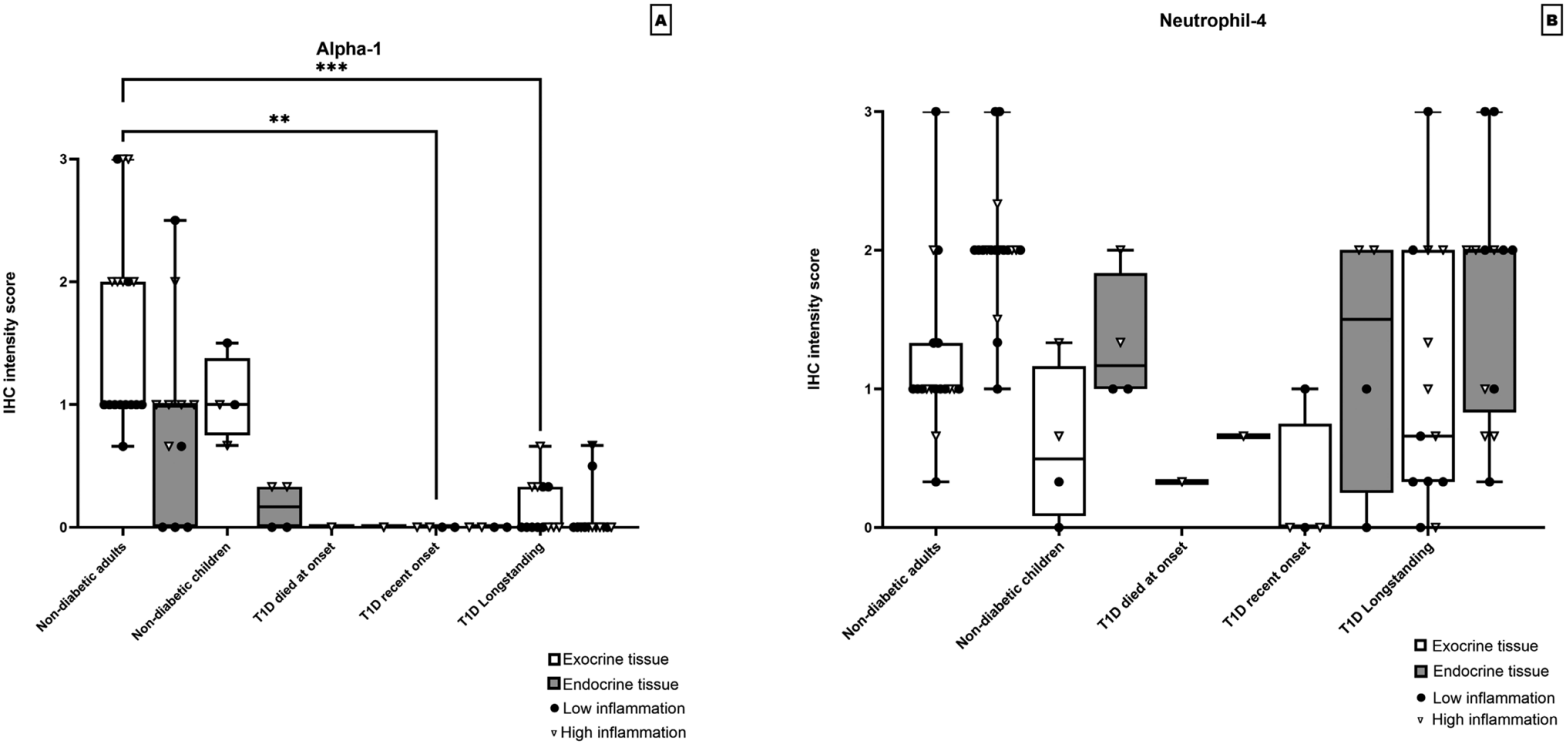
Each data point represents the mean value of the IHC score of the three pancreatic regions in one donor in endocrine and exocrine tissue, respectively. The donors were divided into different groups depending on disease status, non-diabetic adults (*n* = 19), non-diabetic children (*n* = 4), Type 1 diabetes donor that died at onset (*n* = 1), Type 1 diabetes recent onset (DiViD patients) (*n* = 4) and Type 1 diabetes longstanding donors (*n* = 13). The IHC scores for defensin Alpha-1 are shown in **a** and defensin Neutrophil-4 in **b**. Statistically significant differences are shown in each figure using Kruskal–Wallis test and Dunns test for multiple comparisons p values were non-significant > 0.05 for all groups

Defensin Alpha-1 was observed with a patchy cytoplasmatic staining pattern in exocrine parenchyma in the adult non-diabetic control group (Fig. 1a). A dichotomic expression in endocrine tissue was found. Many subjects with no expression and others with a cytoplasmatic and nucleic expression (mean IHC score = 0.94). In the child group the same expression pattern was observed but with a lower grade of staining, mean IHC score of 1 (exocrine tissue), mean IHC score of 0 (endocrine tissue), respectively. Almost all Type 1 diabetes donors were negative (IHC score = 0) in both endocrine and exocrine tissue (Fig. 1b, c). Some donors with longstanding Type 1 diabetes had a low-grade positivity in occasional biopsies (mean IHC score = 0.09). Statistically significant differences were found in both endocrine and exocrine pancreas between non-diabetic adults and subjects with recent onset and longstanding Type 1 diabetes (*p* ≤ 0.05, Fig. 3a). Blood vessels, connective tissue, ducts and adipose tissue were negative for the staining in all donors.

Defensin Neutrophil-4 had a cytoplasmatic expression with great individual variation with a seemingly lower expression in exocrine tissue (mean IHC score = 1.09) when compared with endocrine (mean IHC score = 1.84) tissue (Fig. 3b). Positive granular cytoplasmatic staining, with variation between donors, could also be observed in some exocrine and endocrine cells as well as in blood vessels. Nuclear staining of cells in blood vessels was also observed. Connective tissue, ducts and adipose tissue were negative for the staining in all donors. No statistically significant differences could be observed (*p ≥ *0.05) (Fig. 3b).

### Other defensins

**Fig. 4:**
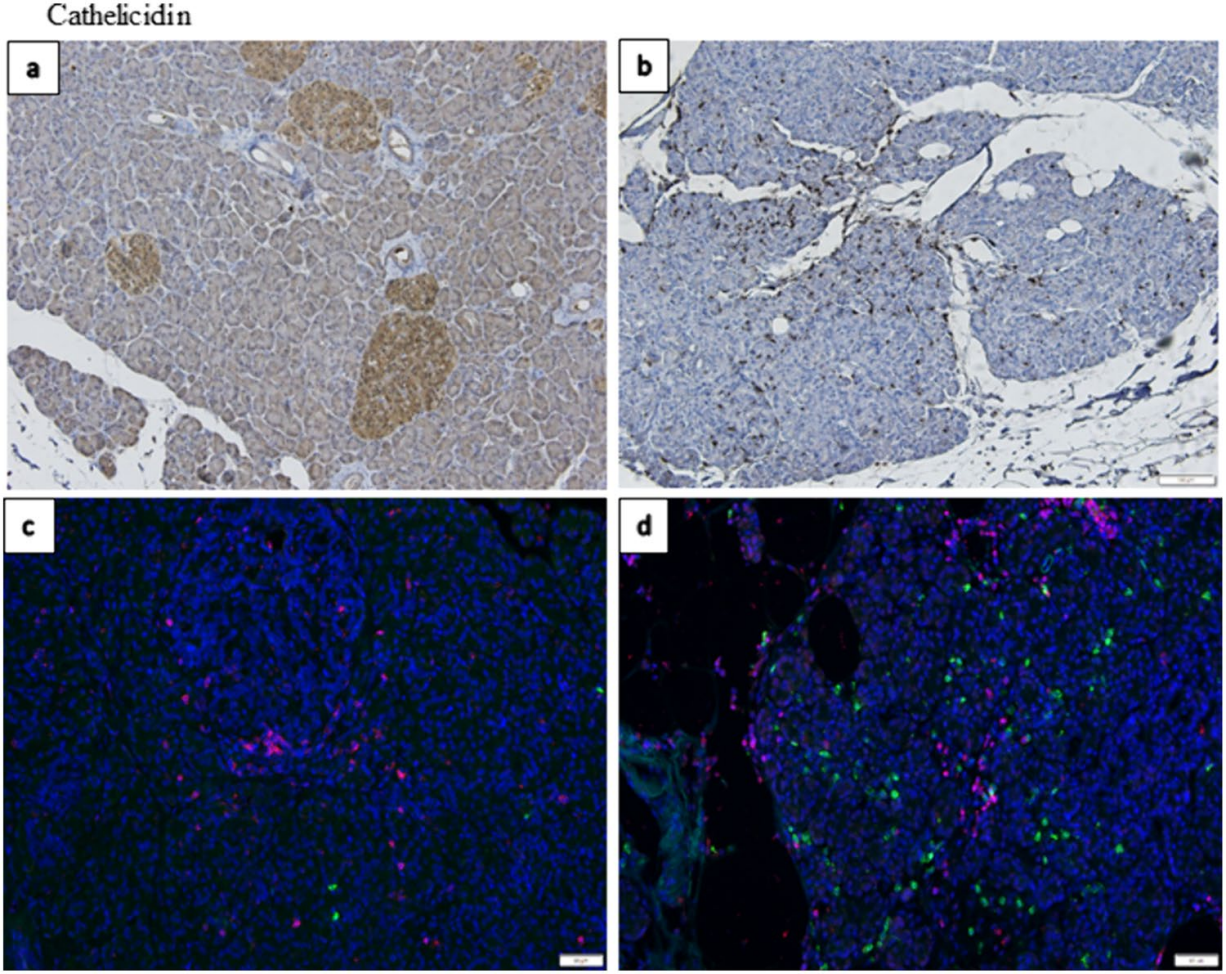
**a** Representative IHC staining of Cathelicidin in an adult without Type 1 diabetes show a cytoplasmatic homogenous staining in both exocrine and endocrine tissue. The staining in the endocrine pancreas was markedly more intense than in the exocrine tissue. **b** No Cathelicidin expression could be detected in the exocrine and endocrine tissue in the donor that died at onset of Type 1 diabetes. However, a large amount of intensely positive stained single cells scattered throughout the pancreatic tissue was observed. **c** Immunofluorescence staining of Cathelicidin (green) and CD3 (red) in a DiViD patient with an islet with insulitis in the center of the slide as well as in a subject with longstanding Type 1 diabetes in **d**  with ongoing inflammation in the pancreas, show no co-staining for Cathelicidin (green) and CD3 (red). Original magnification × 20

Cathelicidin was expressed as a homogenous staining in the cytoplasmic compartment in exocrine tissue in the adult non-diabetic control group (Fig. 4a). Occasional single cells with a more pronounced cytoplasmatic staining were observed in the exocrine tissue. Two control donors showed a high number of this single cell staining. The endocrine tissue was homogeneously stained in the cytoplasmic compartment (mean IHC score = 2.75). The exocrine parenchyma in the child group showed a low-grade cytoplasmic staining (mean IHC score = 0.58), also a few scattered single cells with high level of expression was found in some of the children. In the endocrine tissue the expression varied between donors from IHC score = 0 to a homogenous low-grade cytoplasmic staining (mean IHC score = 0.58).

The donor that died at onset had a negative cytoplasmic expression in both endocrine and exocrine tissue. However, numerous positive single cells in the exocrine tissue was found (Fig. 4b). The recent onset patients stained low-grade positive in the cytoplasmic compartment (mean IHC score = 1) of the exocrine tissue. In donors with positive single cells, the cells were scattered in some but not in other lobes of the pancreas. These cells could also be found accumulating adjacent to some islets. The endocrine tissue was positive in most islets (mean IHC score = 1.75), but negative islets (IHC score = 0) did occur in all four donors.

The staining pattern in subjects with longstanding Type 1 diabetes varied depending on age. In the age group 15–25 years (*n* = 4) the exocrine tissue showed negative or low-grade positive staining in the cytoplasmic compartment (mean IHC score = 0.83). Three donors also had positive single cell staining in the exocrine tissue of varying degrees as described above. The endocrine tissue stained homogenously in the cytoplasmic compartment (mean IHC score = 1).

**Fig. 5:**
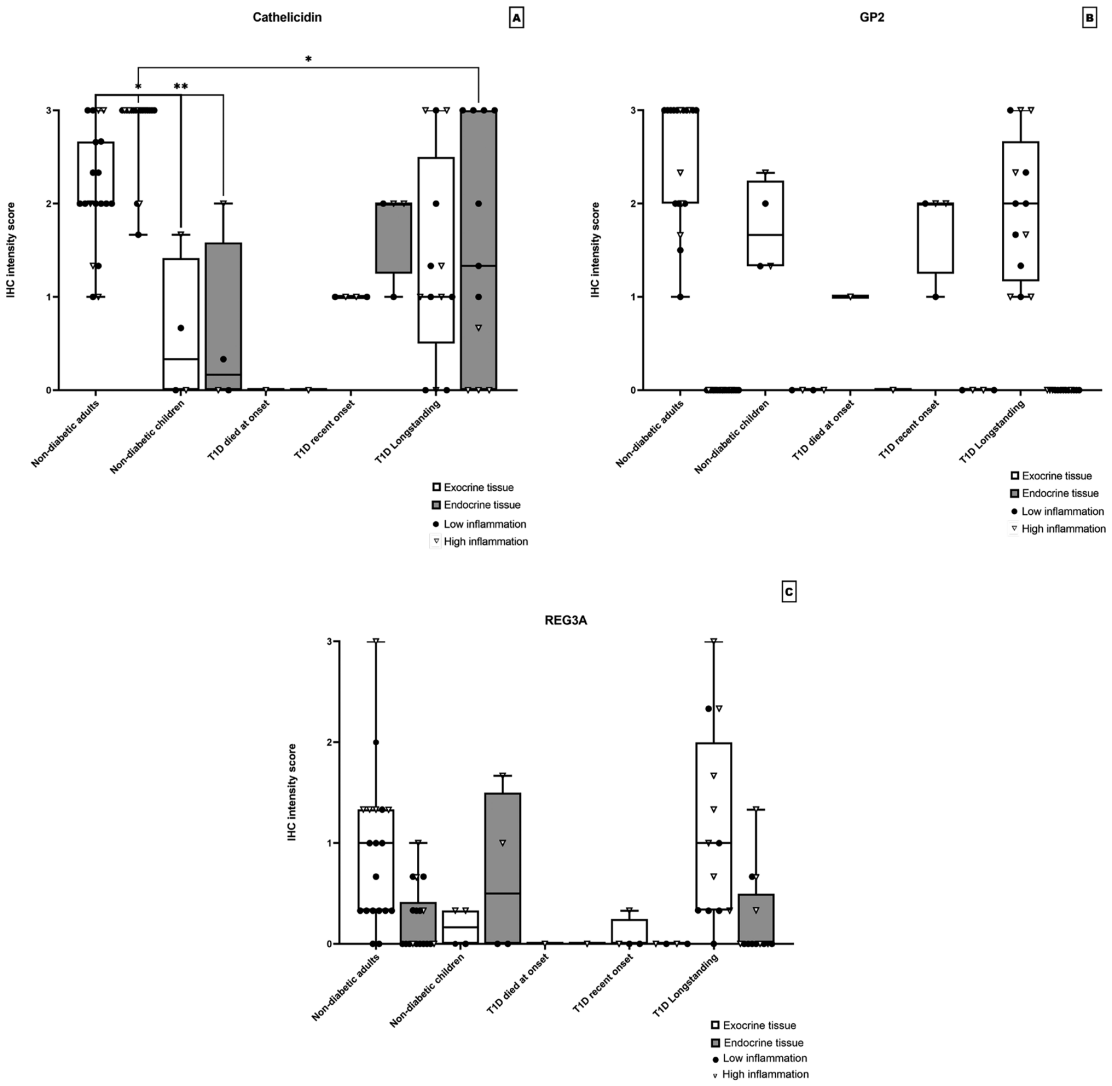
Each data point represents the mean value of the IHC score of the three pancreatic regions in one donor in endocrine and exocrine tissue, respectively. The donors were divided into different groups depending on disease status, non-diabetic adults (*n* = 19), non-diabetic children (*n* = 4), Type 1 diabetes donor that died at onset (*n* = 1), Type 1 diabetes recent onset (DiViD patients) (*n* = 4) and Type 1 diabetes longstanding donors (*n* = 13). The IHC scores for Cathelicidin is shown in **a**, defensin GP2 in **b** and defensin REG3A in **c**. Statistically significant differences are shown in each figure using Kruskal–Wallis test and Dunns test for multiple comparisons p values were non-significant > 0.05 for all groups

In the age group 25–55 years two of the three donors had a homogenous staining of the cytoplasmic compartment (mean IHC score = 1.33) in the exocrine parenchyma and with an even higher intensity in the islets (mean IHC score = 3). Varying numbers of high intensity positive single cells was found in both donors. The third donor was negative in both the exocrine and endocrine parenchyma. Age group 55–70 years all donors exhibited a positive homogenous staining of the cytoplasmic compartment of both exocrine (mean ICH score = 2.25) and endocrine tissue (mean IHC score = 1.66). All donors also displayed varying numbers of scattered positive single cells throughout the tissue. A statistically significant reduction in expression of Cathelicidin in both exocrine and endocrine pancreas were found in children when compared with adults (*p ≤ *0.05, Fig. 5a). Also, expression of Cathelicidin in endocrine pancreas was reduced in subjects with longstanding Type 1 diabetes. No positive staining in blood vessels, connective tissue, ducts or adipose tissue was found in any group.

The positive single cells were further investigated with IF staining for different immune cells. Cathelicidin stained positive in innate immune cells of the pancreas. In the donor that died at onset of Type 1 diabetes the scattered Cathelicidin+ cells co-stained with NES or CD68. No CD3 positive cells were found. In the recent onset group the few scattered Cathelicidin+ cells co-stained with CD68. CD3 cells were present in proximity to islets (insulitis), however no CD3+ cell co-stained with Cathelicidin (Fig. 4c). In subjects with longstanding Type 1 diabetes all the scattered Cathelicidin+ stained cells co-stained with CD68. CD3 positive cells was also present in the older donors but showed no co-staining with Cathelicidin (Fig. 4d).

The pancreatic marker GP2 was observed as a homogenous cytoplasmic staining of the exocrine pancreas in all individuals (mean IHC score = 2.17). However, in children and in subjects with Type 1 diabetes large areas of unstained exocrine tissue were found. Endocrine tissue, blood vessels, connective tissue, ducts and adipose tissue was negative for the staining in all donors. No statistically significant difference could be observed (*p* ≥ 0.05 Fig. 5b).

Defensin REG3A was expressed as a cytoplasmatic staining with a patchy appearance in the exocrine tissue that correlated with the local inflammation, expression in endocrine tissue was low-grade (mean IHC score = 0.25 Fig. 1g–i). In children and in subjects with recent onset Type 1 diabetes the expression was lower in exocrine tissue (mean IHC score = 0.12). In subjects with longstanding Type 1 diabetes the level of expression was slightly higher compared to adult non-diabetic individuals (mean IHC score = 1.02). Blood vessels, connective tissue, ducts and adipose tissue were negative for the staining in all donors. No statistically significant differences could be observed (*p* ≥ 0.05 Fig. 5c).

### Inflammation in correlation to defensin expression

In non-diabetic adult controls the level of expression for defensins Beta-1, Alpha-1, Cathelicidin and REG3A correlated with the level of inflammation (Figs. 2a, 3a, 5a and c). Inflammation in diabetic donors at onset was more intense and affected larger areas of the exocrine pancreas when compared with that observed in non-diabetic subjects. Inflammation consisted mostly of CD45+ cells but also CD3+ cells were found both in the exocrine parenchyma and in the vicinity of islets; peri-insulitis. Even so, most donors with Type 1 diabetes expressed no or only low levels of defensins Beta-1 and Alpha-1 (Figs. 2a and 3a). No significant differences in expression of Cathelicidin were found between non-diabetic and Type 1 diabetic subjects. Except for donors with longstanding Type 1 diabetes whom had a lower expression of Cathelicidin in islets (Fig. 5a). Notably, a majority of these donors with high inflammation in their pancreas showed no expression of Cathelicidin.

## Discussion

Herein we report a markedly reduced expression of central defensins belonging to different families in both exocrine and endocrine tissue of the pancreas in subjects with Type 1 diabetes as compared to non-diabetic controls. Moreover, we found a positive correlation in non-diabetic subjects between inflammation in both endocrine and exocrine tissue and expression of several of the defensins. Notably, this correlation was markedly reduced or even absent in subjects with Type 1 diabetes. Among the three beta-defensins expression of Beta-1 is of most importance against bacterial infections [23]. Beta defensins also modulate the adaptive immune response by promoting chemotactic activities for immature dendritic cells and memory T-cells [7, 24]. Expression of Beta-1 was reduced in subjects with Type 1 diabetes compared to non-diabetic adult controls. Glucose homeostasis seems to have an important role in controlling expression of Beta-1 [23, 25]. However, no difference in expression of Beta-1 could be observed between the Type 1 diabetes groups suggesting a reduced expression of Beta-1 in subjects with Type 1 diabetes per se.

The Alpha defensins are part of a major interplay between healthy gut homeostasis, microbiota and innate immune system. The defensin resides in granules which are released when bacteria is present [1]. Decreased expression of Alpha-defensins cause an imbalance of this intricate system which has been considered as a trigger for inflammatory events and has been reported in Crohn´s disease [26]. Similarly, decreased expression of Alpha-1 defensin in subjects with Type 1 diabetes was found.

Children and subjects with Type 1 diabetes also had a marked reduction in the expression of Cathelicidin when compared with non-diabetic adult controls. With increasing age and therefore longer duration of Type 1 diabetes the expression pattern changed. In the oldest subjects with the longest disease duration expression showed a similar staining pattern as non-diabetic adults. Expression of Cathelicidin was also found in macrophages and neutrophilic granulocytes scattered in the exocrine pancreas especially in the Type 1 diabetes subjects. T-cells in the insulitic lesions in subjects with recent onset Type 1 diabetes were negative. These findings are in agreement with previous reports on expression of Cathelicidin in granules of neutrophils and macrophages [27]. Cathelicidin is not a defensin per se, but it is classified as an antimicrobial peptide and has a wide range of immunomodulatory effects [28]. Expression of Cathelicidin is dependent on vitamin-D [29]. Several studies have implied a correlation between induced risk of Type 1 diabetes and vitamin-D deficiency [30, 31]. Vitamin-D also plays an important role in beta-cell function and development [29, 30, 32, 33].

The defensin REG3A showed a slightly lower expression in children and subjects with recent onset Type 1 diabetes when compared with non-diabetic adults. Notably, subjects with longstanding Type 1 diabetes on the other hand had a slightly higher expression than non-diabetic adults. REG3A has been reported in several studies to have a protective role for beta-cells when overexpressed [34–36]. REG3A has also been shown to be altered by mild hyperglycemia [37]. Even if no statistically significant differences could be found, the findings presented support the view that islets in subjects with Type 1 diabetes suffer from a relative deficiency of REG3A [34–37].

Defensins have an important role in regulating inflammation and acquired immunity. In non-diabetic donors a correlation between pancreatic inflammation and expression of Beta-1, Alpha-1, Cathelicidin andREG3A were found. Inflammation in donors with Type 1 diabetes was more intense and affected larger areas of the exocrine pancreas when compared with that observed in non-diabetic subjects. Even so, expression of defensins remained low. Obtained findings implies that there could be a disturbance in how the innate immune system responds in individuals with Type 1 diabetes, tentatively causing prolonged local inflammation with negative effects on exocrine and endocrine homeostasis. Indeed a patchy inflammation affecting both the exocrine and endocrine pancreas has frequently been reported in subjects with Type 1 diabetes [14–17].

Possible limitations of our study are the relatively low numbers of biopsies available from subjects with newly diagnosed Type 1 diabetes. However, it is well established that this type of biopsies remains rare. Another point of consideration is that the IHC staining allows detailed analysis of the cellular expression pattern of a specific protein, but it is not optimal in quantifying the exact amount of protein. However, for proteins with posttranslational modifications, e.g. defensins, alternative quantitative techniques such as in situ hybridization are not applicable.

Collectively, presented findings implicate a disturbance in the innate immune response in subjects with Type 1 diabetes. Reduced or even lack of expression of defensins could cause prolonged and exaggerated inflammation and dysregulation of the bridge to the adaptive immune responses [7, 24]. A similar reduced expression of defensins has been seen in other inflammatory diseases [4, 7]. The findings presented support an important role for defensins in Type 1 diabetes and further studies on the role of the innate immune system in Type 1 diabetes is needed.

## Data Availability

The data sets generated during the current study are available from the corresponding author upon reasonable request

## Abbreviations

DiViD: Diabetes virus detection
FFPE: Formalin-fixed paraffin-embedded
IF: Immunofluorescence
IHC: Immunohistochemistry
mAb: Monoclonal antibody
HbD-1: Human beta defensin 1
